# Modafinil Induced Psychosis in a Patient with Bipolar 1 Depression

**DOI:** 10.1155/2018/3732958

**Published:** 2018-10-17

**Authors:** Alexander A. DiSciullo, Clayton D. English, William T. Horn

**Affiliations:** ^1^University of Vermont Medical Center, USA; ^2^Albany College of Pharmacy and Health Sciences, USA

## Abstract

Modafinil has been used as an adjunctive medication in the treatment of bipolar 1 depression with reported success. Case reports have been published demonstrating modafinil induced mania in bipolar patients and modafinil induced psychosis in schizophrenic patients. To our knowledge, we report the only case of modafinil induced psychosis in a patient with bipolar depression treated with both mood stabilizers and antipsychotics. In addition, it is the quickest onset to psychosis (2 days) at the lowest dosage of modafinil (100 mg/day) reported in the literature. Although favorable outcomes using modafinil for treatment of bipolar depression have been reported in literature, clinicians should remain cautious of the potential to rapidly induce psychosis with modafinil at low dosages in patients with bipolar depression despite being treated with mood stabilizers and antipsychotics.

## 1. Introduction

Modafinil is typically used to induce wakefulness in the treatment of narcolepsy and obstructive sleep apnea [1–3]. More recently, it has been used as an adjunctive medication in the treatment of bipolar 1 depression with reported success. Several double-blinded, placebo-controlled trials of modafinil use in bipolar depression have shown significant improvements in baseline to endpoint change in score on the Inventory of Depressive Symptoms score (IDS) in modafinil groups when compared to placebo [4, 5]. Other studies have showed that adjunctive modafinil improves severe hypersomnia, depressive symptoms, and patient functioning with a favorable side effect profile in bipolar depression patients on mood stabilizers [6, 7]. Canadian Network for Mood and Anxiety Treatments (CANMAT) guidelines currently have adjunctive modafinil listed as a second line combination therapy for the treatment of bipolar 1 depression [8].

However, multiple case reports have been published which have demonstrated modafinil induced mania at various dosages in patients with bipolar disorder [9–13]. Further there have been reports of modafinil induced psychosis in patients with schizophrenia, Dementia with Lewy Bodies, and in patients with excessive daytime sleepiness and difficulty concentrating [14–18].

We report a case of rapid modafinil induced psychosis at a low dosage in a patient with bipolar 1 disorder with severe depression and hypersomnia.

## 2. Case Report

This patient was a 48-year-old currently employed male with a diagnosis of bipolar 1 disorder who was admitted to our inpatient psychiatric unit for treatment of severe bipolar 1 depression. About two months prior to this admission for depression, he had been involuntarily hospitalized at another facility for mania.

Standard laboratory measures, which were within normal limits, and a urine toxicology screen, which was negative, were obtained prior to admission. During the initial days on our service, the patient endorsed depressed mood and low energy. He had profound hypersomnia and slept through the night and much of the day. He rarely would attend group therapy or socialize with staff or other patients.

The patient was started on modafinil 100 mg daily with plans to use short term to help combat hypersomnia. Psychiatric medications at the time included divalproex 2,500 mg QHS, quetiapine 300 mg QHS, and venlafaxine 225 mg once daily. Venlafaxine had been increased to 225 mg several weeks prior to initiation of modafinil. His valproic acid level prior to initiation of modafinil was found to be 79 ug/ml, confirming adequate prophylactic treatment of mania. There were no other changes made to his medication regimen at this time. Two days following the initiation of modafinil the patient begins to demonstrate symptoms of psychosis. This included seeing trees moving in his bedroom, beliefs that there were cameras in the pictures on his wall, and that a water bottle was “transmitting something” into his room. The following day the patient demonstrated more psychotic behaviors including waking his roommate up in the middle of the night to accuse his roommate of abusing his daughter and later accusing the treatment team of including him in experimental research. Following these psychotic events, the modafinil was discontinued and the psychotic features subsided within the following days.

## 3. Discussion

To our knowledge this is the only case report of modafinil induced psychosis in a patient with bipolar depression taking both mood stabilizers and antipsychotics. In addition, it is the quickest onset to psychosis (2 days) at the lowest dosage of modafinil (100 mg/day) reported in the literature.

Although the time course of initiation of modafinil correlates with the onset of psychosis, it is possible that the combination of both modafinil and venlafaxine could have induced psychosis due to synergistic effects and clinicians should be aware of this potential interaction. Secondly, it is important to note that hypersomnia is an atypical feature of bipolar depression and it is possible that this clinical subgroup of patients is more vulnerable to modafinil induced psychosis than patients without atypical features.

The mechanism of action of modafinil, in addition to its potential mechanism of inducing psychosis, is unknown. It has been proposed that modafinil may inhibit the release of y-aminobutyric acid (GABA) resulting in a loss of inhibition of the excitatory cholinergic and glutaminergic pathways resulting in psychosis [19, 20]. It has also been proposed that modafinil increases dopamine levels via inhibition of GABA release and via weak inhibition of dopamine reuptake thereby potentiating psychosis [13, 20]. More research needs to be conducted regarding the mechanisms of action of modafinil and its potential to induce psychosis.

Although favorable outcomes using modafinil for treatment of bipolar depression have been reported in literature, clinicians should remain cautious of the potential to rapidly induce psychosis with modafinil at low dosages in patients with bipolar depression despite being treated with mood stabilizers and antipsychotics.
